# Reforming care without bureaucracy: Commentary on… Care Programme Approach

**DOI:** 10.1192/bjb.2018.69

**Published:** 2019-06

**Authors:** Peter Tyrer

**Affiliations:** 1 Centre for Psychiatry, Imperial College, London, UK

**Keywords:** Community care, Care Programme Approach, severe mental illness, government policy

## Abstract

The Care Programme Approach was a valiant attempt to improve the aftercare of people with severe mental illness after discharge from hospital. It was introduced as a response to a scandal, not an advance in knowledge, and has always suffered by being a reaction to events rather than a trailblazer for the future. It may have dragged the worst of care upwards, but at the expense of creating a bureaucratic monstrosity that has hindered good practice by excessive attention to risk, and vastly increased paperwork with intangible benefit. It needs to be simplified to allow practitioners greater scope for collaborative solutions, less minatory oversight and better use of strained resources.

**Declaration of interest:**

None.

The article by David Kingdon,^1^ who is over-modest about his own role in creating the Care Programme Approach (CPA) for mental health services, is a welcome suggestion to a system that needs changing. To understand the CPA it is necessary to understand the full history behind it. As David Kingdon indicates, it came about not following new research evidence but – like most reforms in the National Health Service (NHS) – through scandal, and all should be sceptical about its benefits under these circumstances until independent evidence becomes available.

So picture the situation in 1988, when the Spokes Inquiry^2^ made recommendations following the murder of a social worker by an ex-patient in her offices in Bexley. The patient, Sharon Campbell, had apparently been discharged without adequate supervision and the report sent a shudder through the corridors of the NHS hierarchy and the Royal College of Psychiatrists. How many other potentially dangerous patients were similarly being treated, or even untreated, as a consequence of the decision to close hospitals and move towards community care?

## The Spokes Inquiry

The Spokes Inquiry^2^ made appropriate and reasonable recommendations that could be summarised as ‘when psychiatric patients, particularly those with severe mental illness, are discharged from hospital there needs to be an after-care plan in place for both health and social services’. The implementation of these plans was not specified exactly and the Royal College of Psychiatrists was asked to create such an aftercare policy. There already was a system called case management, which had devotees, but most of the evidence was imported unwisely from the USA where the notion of universal care was anathema to the land of the free market.

So the CPA was introduced as a diluted form of case management appropriate for the NHS. Note the wording. It was not a mandatory requirement for practitioners to do this or that, but a gentle nudge to ensure a coordinated system of care, an approach rather than a directive. It certainly worked to a point; consultants could no longer discharge patients to follow-up by the general practitioner without some sort of care plan in place. The CPA was introduced so gently that its implementation was almost imperceptible, allowing a randomised trial to take place using the old system of care as the comparator. The results showed that many fewer patients were lost to follow-up, but readmissions were much more common once good follow-up was in place.^3^

## Early years of the CPA

The notion of care plans – and the need for a single person, the care coordinator, to synthesise care with the parties involved – is a sensible one and many health professionals felt they were doing it already. It was held together successfully at first through the efficiency and cooperation of community psychiatric teams, whose contribution and value, including a reduction in deaths,^4^ has been somewhat underestimated as these teams have never had the glamour of assertive outreach and crisis resolution teams. Where it began to fail was not a fault of the approach but a lack of resources to implement it properly, so it was never applied universally. It is very odd that no organisation within the healthcare system made any attempt to cost the full implementation of the CPA. What may also not have been anticipated was the rapid growth of managers once cost pressures increased,^5^ and whose attempts to improve integration of care were often resented by practitioners as undue interference.^6^

Thus, 15 years after its introduction, Simpson *et al*^7^ concluded after a careful review in 2003:

‘The CPA was a flawed policy introduced insensitively into an inhospitable environment. It was destined to fail and after more than a decade remains ineffectively implemented. Changes introduced recently may have contradictory influences on the ability of services to provide effective case management but remain to be evaluated.’^7^

## Standard and Enhanced CPA

In 2008 the Enhanced CPA was subsequently introduced for people ‘who need: multi-agency support; active engagement; intense intervention; support with dual diagnoses; and who are at higher risk’.^8^ It was also emphasised that acceptance for enhanced care ‘should *not* be used as a “gateway” to social services or as a “badge” of entitlement’^8^ to other services. But of course it was used that way, as it was bound to be; thus it merely added another layer of bureaucracy to an over-burdened system.

The consequence has been an increasing transfer of face-to-face clinical care to a paperchase cynical affair that does no credit to anybody, least of all the patients, who all too frequently see a technocrat facing a desk instead of a sympathetic carer across the room. The tick box has now become the kick box, an exercise to get a patient off one team's case load and on to another, promoting discontinuity and disruption of care.

## What is the solution?

There are many lessons to be learned about the CPA. First, it has to be accepted by all health professionals, politicians and society at large that suicides and violent deaths in the community perpetrated by psychiatric patients can never be prevented altogether. Putting this another way: extreme statistical outliers should not determine policy. Second, and probably most importantly, good psychiatric and social care is flexible and collaborative and can never be prescribed by statute. I recently discharged myself from hospital prematurely against medical advice. But I insisted the record was not recorded as ‘against advice’ by writing in detail in the hospital notes why it was better for me to leave hospital as my aftercare was well arranged and would lead to cost savings. (They accepted this and were probably pleased to see me go.)

Third, allow practitioners and patients, working together, to do what is best and not to be too risk averse. Kingdon recommends that we should develop ‘more individualised and sophisticated pathway-based systems’.^1^ This is in itself a telling criticism of the CPA. Once we have such individualised systems, we leave the directives behind; we use a combination of skills and resources to produce a plan that is, more often than not, unique and cannot be classified. Such plans can never be truly evidence based.

So, in summary, the CPA needs reform by becoming simpler rather than more complicated. It is there to prevent poor care, not to interfere with care that is already competent or good. Its wording should be chosen with care to allow good clinicians to be reinforced and praised in their tasks, for nervous ones to be encouraged and for those who are under par to receive a gentle rap on the knuckles to improve their game. It can also be a great deal shorter.

## About the author

**Peter Tyrer** is Emeritus Professor of Psychiatry at Imperial College, London, UK.
